# An Approach for Integrating the Prioritization of Functional and Nonfunctional Requirements

**DOI:** 10.1155/2014/737626

**Published:** 2014-04-10

**Authors:** Mohammad Dabbagh, Sai Peck Lee

**Affiliations:** Faculty of Computer Science and Information Technology, University of Malaya, Kuala Lumpur 50603, Malaysia

## Abstract

Due to the budgetary deadlines and time to market constraints, it is essential to prioritize software requirements. The outcome of requirements prioritization is an ordering of requirements which need to be considered first during the software development process. To achieve a high quality software system, both functional and nonfunctional requirements must be taken into consideration during the prioritization process. Although several requirements prioritization methods have been proposed so far, no particular method or approach is presented to consider both functional and nonfunctional requirements during the prioritization stage. In this paper, we propose an approach which aims to integrate the process of prioritizing functional and nonfunctional requirements. The outcome of applying the proposed approach produces two separate prioritized lists of functional and non-functional requirements. The effectiveness of the proposed approach has been evaluated through an empirical experiment aimed at comparing the approach with the two state-of-the-art-based approaches, analytic hierarchy process (AHP) and hybrid assessment method (HAM). Results show that our proposed approach outperforms AHP and HAM in terms of actual time-consumption while preserving the quality of the results obtained by our proposed approach at a high level of agreement in comparison with the results produced by the other two approaches.

## 1. Introduction

The ultimate goal of developing any software system is to satisfy various stakeholders' needs [1]. Hence, managing the software requirements process plays a critical role towards the success of a software development project [2]. Requirements engineering, as a first step in the software development process, and its underlying activities can help practitioners to understand stakeholders' needs and develop high quality software in an economic manner.

However, due to the budgetary deadlines and time to market constraints, it could be a challenge for requirements engineers to decide which requirements lead to high stakeholder satisfaction and need to be considered first. To address this concern and in order to reduce the cost and duration of a software project as well, it is essential to address the high-priority requirements before considering the low-priority ones [2–4]. Requirements prioritization can help to identify the most important requirements for a software system [5] and then proceed to develop the software according to these requirements. Hence, requirements prioritization has been recognized as one of the most important decision making processes during the software development process [1, 6, 7].

The requirements engineering community has classified the requirements of a software system into two main categories: functional requirements and nonfunctional requirements [8]. Functional requirements describe the functional behavior of the system whereas nonfunctional requirements express how good a system should work. It has been widely acknowledged that a quality attribute such as reliability, modifiability, performance, or usability is a nonfunctional requirement of a software system [8–10]. Although functional and nonfunctional requirements are very different, they have a serious impact on each other. Several studies, for example, [11–15], stated that the achievement of nonfunctional requirements along with functional requirements is critical to the success of a software system. Hence, prioritizing these two types of requirements entirely together or separately might not be the best solution [16]. For example, if there is one functional requirement about a specific function and one nonfunctional requirement regarding availability, it could be hard to prioritize between them since they are not at the same abstraction level. So, it is not an efficient way to prioritize both types of requirements together. In such cases, one may decide to prioritize them separately. Clearly, it is also not a good choice since both types of requirements have an impression on each other.

Numerous methods on requirements prioritization have been introduced in recent years, the most widely known of which being analytic hierarchy process (AHP) [17], cost-value approach [1], Wieger's method [18], and value-oriented requirements prioritization (VOP) [19], and more recently there is a method which applied interactive genetic algorithm to prioritize requirements [20]. Although these methods have contributed a lot to software development process and can be used with both kinds of requirements separately (these methods have mostly been adopted just with functional requirements [14, 21]), there is a need for proposing an approach in which prioritization of functional and nonfunctional requirements could be integrated. This paper is primarily concerned with providing such an approach.

In this paper, we introduce integrated prioritization approach (IPA), an approach which prioritizes both functional and nonfunctional requirements simultaneously, producing two prioritized lists of functional requirements and nonfunctional requirements separately. The key contribution of IPA over existing work is to provide a requirements prioritization framework which considers both functional and nonfunctional requirements during the prioritization process. One major characteristic of IPA is that it requires only one decision matrix to perform the prioritization task. In this paper, we have used the term nonfunctional requirements and quality attributes interchangeably to mean the same. Furthermore, in this paper, we describe an empirical study which has been conducted to compare the IPA with the two state-of-the-art-based approaches, analytic hierarchy process (AHP) and hybrid assessment method (HAM).

The remainder of this paper is organized as follows. Requirements prioritization methods from the literature are introduced in Section 2. The proposed approach for integrating the prioritization of functional and nonfunctional requirements and its supporting tool along with an illustrative example are presented in Section 3. The detailed description of the experiment which has been carried out during this study is presented in Section 4. Finally, Section 5 concludes and outlines future work.

## 2. Related Work

As the complexity of software systems increases, practitioners are forced to make trade-offs between conflicting requirements in order to complete projects on predefined schedule. Priority assessment of requirements is one of the techniques which can be useful to assist practitioners to resolve trade-offs. Thus, requirements prioritization has become an increasingly important part of ensuring the success of a project, and, consequently, various works pointed out the importance of the problem of requirements prioritization in the software engineering domain.

Even though prioritization techniques have mostly been adopted with respect to functional requirements [14, 21], several studies have shown the significance of nonfunctional requirements in software projects [22–25], and not correctly taking nonfunctional requirements into consideration is identified as one of the ten biggest risks in requirements engineering [26]. Therefore, nonfunctional requirements need to be considered throughout the first phase of the software development process (i.e., requirements engineering phase) and it has been acknowledged that the achievement of nonfunctional requirements along with functional requirements is critical to the success of a software system [11–15]. All these reasons motivated us to concentrate on both functional and nonfunctional requirements in this study and propose an approach which considers these two types of requirements simultaneously during the prioritization process.

A rich literature on requirements prioritization is available in the research literature. It includes studies that investigate the requirements prioritization role in software development processes, proposing a number of approaches to carry out requirements prioritization and a growing set of empirical studies dedicated to evaluations giving an account of the benefits and drawbacks of applying a particular approach. Focusing on requirements prioritization approaches, they can be split into two categories, depending on the order relation they ultimately produced. The rank assigned to requirements by a prioritization approach may define either a total ordering of the requirements or a partial ordering. Having a total ordering could simplify the allocation of requirements to the next release. Our work belongs to the first category. In the following, some of the important ones are explained briefly.

One of the powerful and flexible techniques which has been widely used to prioritize requirements is the analytic hierarchy process (AHP), first introduced by Saaty [17]. In AHP, all pairs of requirements are compared together to determine the priority level of one requirement over another requirement. By applying AHP, all requirements are first put in the rows and columns of a matrix. Then, the user specifies his/her priority to each pair of requirements by assigning a preference value which is between 1 and 9, where 1 expresses equal value while 9 indicates extreme value. After that, AHP converts these scales to numerical values, and, consequently, a numerical priority is derived for each requirement. There are also some variations of AHP that have been proposed by researchers, such as case-based ranking (CBRank) [27] which adopts a preference elicitation process that combines sets of preferences elicited from human decision makers with sets of constraints which are automatically computed through machine learning techniques. It also exploits knowledge about partial rankings of the requirements that may be encoded in the description of the requirements themselves as requirement attributes (e.g., priorities or preferences). Cost-value approach [1] is another AHP-based approach which prioritizes requirements based on two aspects: importance of requirement to customers and cost of implementing each requirement. In the research work presented in [15], the other AHP-based approach has been proposed to prioritize nonfunctional requirements, contentrating on the internal relationships which exist among the candidate list of nonfunctional requirements.

In [18], Wiegers presents a framework in which requirements are prioritized based on four criteria defined as benefit, penalty, cost, and risk. The input values of these criteria are assessed on a scale from 1 (minimum) to 9 (maximum). The customer representative determines the benefit and penalty values whereas the development representative provides the cost and risk values associated with each requirement. Then, by using a formula, the relative importance value of each requirement is calculated. Azar et al. [19] describe a value-oriented prioritization (VOP) framework where requirements are linked to business values and prioritized based on the stakeholder ratings. VOP uses the relationships that exist between core business values to assess and prioritize relative relationships among those values and requirements and ensure their traceability. Company executives identify the core business values and use a simple ordinal scale to weight them according to their importance to the organization. Based on the data, a prioritization matrix is then constructed. In [4], a correlation-based priority assessment is proposed which prioritizes software process requirements gathered from multiple stakeholders by incorporating interperspective relationships of requirements. In [3], a technique is presented for partially automating the prioritization of requirements and raw feature requests in large-scale elicitation processes. Another approach in [20] applied genetic algorithm for prioritizing requirements in order to make prioritization scalable by reducing the number of pairwise comparisons. However, no particular method or approach has been proposed for integrating the prioritization of functional and nonfunctional requirements.

## 3. The Proposed Approach

Our approach for prioritizing functional and nonfunctional requirements assists practitioners to obtain a prioritized list of nonfunctional requirements (NFRs) along with a prioritized list of functional requirements (FRs). According to Berander and Andrews [16], nonfunctional requirements affect several functional requirements, from one to all FRs of a software system. Inspired by this point, we try to find out the extent in which each NFR may affect a given FR. By determining such a value (i.e., the importance degree of an NFR for a given FR), our approach intends to prioritize FRs and NFRs simultaneously, using only one decision matrix.

The framework produces a prioritized list of NFRs by calculating the total importance degree of each NFR with respect to all FRs. In other words, it means that an NFR which achieves the greatest total importance degree among all FRs may be assigned as a high-priority NFR with respect to all FRs. Furthermore, it provides a prioritized list of FRs according to the importance degrees of NFRs.

### 3.1. Integrated Prioritization Approach (IPA)

**Figure 1:**
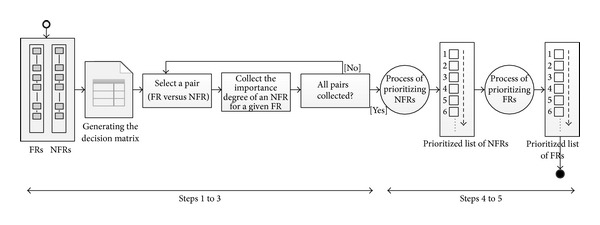
The integrated process of generating the prioritized lists of functional and nonfunctional requirements.

In order to integrate the prioritization of functional and nonfunctional requirements, we proposed an approach, namely, integrated prioritization approach (IPA), consisting of five steps, as shown in Table 1. The first step is to elicit functional requirements as well as nonfunctional requirements. The second step is to set up the *n* functional requirements and the *m* nonfunctional requirements in the rows and columns of an *n* × *m* decision matrix, respectively. The third step performs the elicitation of the importance degree of each NFR with respect to each FR. This step is the only step that requires the decision maker input. The fourth step is an aggregation process to determine the prioritization of NFRs ranking with respect to all FRs using triangular fuzzy numbers and alpha cut approach. This step provides a decision maker with a prioritized list of NFRs with respect to all FRs. The fifth and final step concludes our process by calculating the FRs priority vector and the respective normalized weights.  Figure 1 depicts the overall process.

As can be observed in Table 1, using IPA to prioritize FRs and NFRs involves five steps. The detailed explanation of these steps is illustrated in the following.

**Step 1 (determine candidate FRs and NFRs)**

The first step of IPA is to identify the FRs and NFRs which are required to be prioritized for inclusion in a software system. Let *n* be the number of candidate FRs and *m* the number of candidate NFRs. For demonstration of the process, we assume that there are four candidate functional requirements: FR1, FR2, FR3, and FR4, and three nonfunctional requirements: NFR1, NFR2, and NFR3, which need to be ranked using IPA.

**Step 2 (construct the decision matrix)**

The second step is to generate an *n* × *m* decision matrix, namely, *D*, and insert the *n* functional requirements along with *m* nonfunctional requirements in the rows and columns of the decision matrix, respectively. Following the example stated in the previous step, a 4 × 3 matrix is constructed, as shown in (1). The instructions on how to fill up the elements of the matrix *D* will be described in Step 3

(1) $$\begin{array}{c} D=\begin{array}{cccc} & \text{NFR}1 & \text{NFR}2 & \text{NFR}3\\ \text{FR}1 & - & - & -\\ \text{FR}2 & - & - & -\\ \text{FR}3 & - & - & -\\ \text{FR}4 & - & - & - \end{array} \end{array}$$

**Step 3 (elicit the importance degree of each NFR with respect to each FR)**

The third step elicits the decision maker judgements for determining the importance degree of each NFR for a given FR. To elicit such an extent, the IPA uses two scales: nominal scale and actual scale. Nominal scale is an interface scale which is utilized in order to enhance the user-friendliness of IPA for interacting with decision makers so that the decision maker does not have to know details about the actual scale. On the other side, the actual scale is a numerical scale which is used for internal calculations within IPA. In fact, IPA exploits a five-point scale as actual scale.  Table 2 demonstrates these scales.

Given a pair of FR and NFR (selected from rows and columns of matrix *D*, resp.), the decision maker performs a set of activities aiming at determining the importance degree of each NFR for achieving each associated FR (see activities “select a pair” and “collect the importance degree of an NFR for a given FR” in Figure 1). Each pair (i.e., FR and NFR) is assigned a value belonging to the IPA nominal scale (see left column of Table 2) which represents a qualitative measure of importance relation between the corresponding requirements. So, for all *i* and *j*, with 1 ≤ *i* ≤ *n* and 1 ≤ *j* ≤ *m*, the importance degree of the *j*th nonfunctional requirement for achieving the *i*th functional requirement would be assessed by a decision maker, leading to the value *D*_*ij*_ using Table 2 (e.g., if the value of *D*_23_ = “very high importance,” it means that the decision maker believed that the nonfunctional requirement NFR3 has “very high importance” degree for achieving functional requirement FR2).

**Step 4 (calculate NFRs final ranking with respect to all FRs using triangular fuzzy numbers and alpha cut approach)**

When all pairs have been evaluated, IPA carries out the priority assessment of NFRs with respect to all FRs, which represents the weights for Step 5, using triangular fuzzy numbers and alpha cut approach. In fact, during this step, IPA creates a prioritized list of NFRs through calculating the total importance degree of each NFR with respect to all FRs. The rational behind this idea is that an NFR which achieves the highest total importance degree among all FRs could be assigned as being a high-priority NFR. The following substeps illustrate a stepwise process of computing the priority vector of NFRs.

**Substep 1 (convert the elements of matrix *D* into numerical values)**

First, IPA converts all values of the decision matrix *D*, which were specified according to the nominal scale, into corresponding actual scales, resulting in the matrix $\overset{´}{D}$.

**Substep 2 (set up triangular fuzzy number)**

In order to aggregate the different importance degrees of each NFR for different FRs, the triangular fuzzy number (TFN) is calculated. TFN is capable of aggregating the subjective opinions of a decision maker through fuzzy set theory. In this study, we applied TFN since it is the most popular fuzzy number among the various shapes of fuzzy numbers. The triangular fuzzy number *T*_*x*_*i*__ is represented using the following equations:

(2) Txi=(Lxi,Mxi,Hxi),i=1,…,m, Lxi,Mxi,Hxi∈[0.001,1]

(3) $$\begin{array}{c} M_{x_{i}}=\sqrt[{n}]{D_{x_{i}a}\cdot D_{x_{i}b}\cdot D_{x_{i}c}\cdots D_{x_{i}n}}, \end{array}$$

where *T*_*x*_*i*__ indicates the triangular fuzzy number of nonfunctional requirement “*x*_*i*_”; *L*_*x*_*i*__ and *H*_*x*_*i*__ represent the lowest and highest value of nonfunctional requirement “*x*_*i*_,” respectively; *M*_*x*_*i*__ is generated by calculating the geometric mean of all values belonging to the nonfunctional requirement “*x*_*i*_”; *m* is the total number of NFRs; *n* is the total number of FRs; and *D*_*x*_*i*_*a*_ specifies an opinion of a decision maker toward the importance degree of the nonfunctional requirement “*x*_*i*_” for achieving the functional requirement “*a*.”

**Substep 3 (constructing the fuzzy priority vector)**

**Figure 2:**
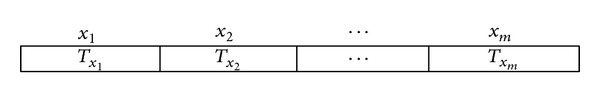
Fuzzy priority vector, ${\tilde{F}}_{x}$.

After calculating the TFN value for each NFR, the fuzzy priority vector, namely, ${\tilde{F}}_{x}$, is generated, as illustrated in Figure 2. Notice that the values of ${\tilde{F}}_{x}$ are derived from (2).

**Substep 4 (defuzzification process)**

IPA exploits the alpha cut approach, proposed by Lious and Wang [28], as shown in (4), to perform the defuzzification process. The defuzzification is accomplished in order to convert the calculated TFN values into quantifiable values, leading to the priority vector *W*

(4) μα,β(F~xi)=[β×fα(Lxi)+(1−β)×fα(Hxi)],0≤α, β≤1,

where *f*_*α*_(*L*_*x*_*i*__) = (*M*_*x*_*i*__ − *L*_*x*_*i*__) × *α* + *L*_*x*_*i*__, which represents the left-end boundary value of alpha cut for ${\tilde{F}}_{x_{i}}$, and *f*_*α*_(*H*_*x*_*i*__) = *H*_*x*_*i*__ − (*H*_*x*_*i*__ − *M*_*x*_*i*__) × *α*, which indicates right-end boundary value of alpha cut for ${\tilde{F}}_{x_{i}}$.

In this context, *α* and *β* carry the meaning of preferences and risk tolerance of decision maker, respectively. These two values range between 0 and 1, in such a way that a lesser value indicates greater uncertainty in decision making. Since preferences and risk tolerance are not the focus of this paper, a value of 0.5 is used for both *α* and *β* to represent a balance environment. This indicates that the decision maker is neither extremely optimistic nor pessimistic about his/her judgments.

Finally, by normalizing the calculated priority vector, *W*, the vector NW of normalized weights is obtained using the following equation:

(5) $$\begin{array}{c} {\text{NW}}_{j}=\frac{W_{j}}{\sum_{j=1}^{m}W_{j}}. \end{array}$$

By applying the steps stated above, a decision maker is provided with a prioritized list of NFRs along with their corresponding importance values with respect to all existing FRs.

**Step 5 (compute FRs final ranking using weighted average decision matrix and weights determined in Step 4)**

During the previous steps, we obtained the priority value of each NFR with respect to all FRs (i.e., NW in Step 4). Furthermore, the importance degree of each NFR regarding every individual FR was elicited (i.e., elements of the matrix $\overset{´}{D}$). By gathering such data, the weighted average decision matrix, as indicated in Table 3, is generated in order to assist the process of calculating the priority vector of FRs (according to their relations with NFRs).

The aggregation method to determine the prioritization of functional requirements in the fifth step of the IPA process is again the calculation of the geometric means but using the obtained normalized priority vector of NFRs (see Substep 4) for its weights, leading to the priority vector *R*, as represented using the following equation:

(6) $$\begin{array}{c} R_{i}=\overset{m}{\underset{1}{\prod }}{{\overset{´}{D}}_{ij}}^{{\text{NW}}_{j}},\;i=1,\ldots ,n. \end{array}$$

Then, the obtained vector *R* is normalized, giving the normalized priority vector of functional requirements, NR, to ensure that the final ranking values will be between 0 and 1:

(7) $$\begin{array}{c} {\text{NR}}_{i}=\frac{r_{i}}{\sum r_{i}}. \end{array}$$

The decreasing ordered functional requirements indicate the final ranking, where the most important functional requirement is the one with the highest NR value.

### 3.2. Example

An intuitive comprehension of the proposed approach can be achieved by applying the IPA to an example, step by step, to demonstrate how the five steps of the IPA could be utilized for a prioritization problem.

**Step 1**

Let us consider a prioritization problem defined over a set of four functional requirements FRs = {FR1, FR2, FR3, FR4} and three nonfunctional requirements NFRs = {NFR1, NFR2, NFR3}, as well.

**Step 2**

In this step, a decision matrix of 4 × 3 is constructed where the rows correspond to functional requirements and the columns correspond to nonfunctional requirements.

**Step 3**

To fill up the elements of the decision matrix, the judgments of a decision maker are elicited and inserted into the matrix, as indicated in (8). In the following, the decision matrix is filled up with nominal scale values:

(8) $$\begin{array}{c} D=\begin{array}{cccc} & \text{NFR}1 & \text{NFR}2 & \text{NFR}3\\ \text{FR}1 & \text{VHI} & \text{HI} & \text{VLI}\\ \text{FR}2 & \text{HI} & \text{VLI} & \text{VLI}\\ \text{FR}3 & \text{LI} & \text{VHI} & \text{HI}\\ \text{FR}4 & \text{HI} & \text{VHI} & \text{HI} \end{array} \end{array}$$

**Step 4**

To calculate the priority vector of NFRs with respect to all FRs, the elements of matrix *D* are converted to actual scales (Substep 1), the TFN is calculated for each NFR (Substep 2), the fuzzy priority vector is constructed (Substep 3), and the defuzzification is done in order to achieve the priority vectors *W* and NW (Substep 4).  Table 4 shows this process where the elements of matrix *D* with actual scales are indicated on the left side while the values of ${\tilde{F}}_{x}$, *W* (4), and NW (5) related to each NFR are represented on the right side of Table 4. By calculating the priority vector NW, we are provided with a prioritized list of NFRs (numbers within parenthesis represent the priority of each NFR for considering during the development process).

**Step 5**

In this phase, the ratings (6) and the normalized ratings (7) for each FR are calculated (see Table 5), using the values of $\overset{´}{D}$ as well as NW which was achieved in Table 4. This step applies a classical weighted average matrix, where rows depict functional requirements while columns depict nonfunctional requirements. By performing this step, the prioritized list of FRs is also achieved (numbers within parenthesis represent the priority of each FR).

### 3.3. Tool-Supported IPA (TIPA)

**Figure 3:**
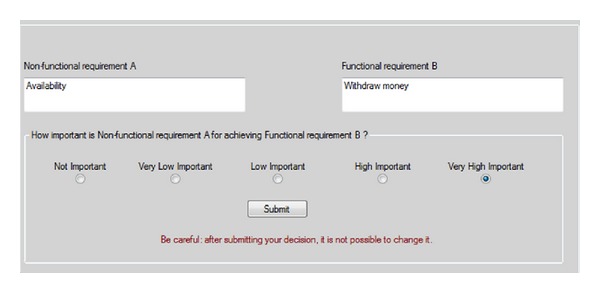
A snapshot of the graphical user interface showing a pair of requirements (availability and withdraw money) under evaluation in TIPA.

The IPA approach is supported by a software tool called tool-supported integrated prioritization approach (TIPA) which allows automating the steps of the IPA process shown in Figure 1. The tool guides the user to apply his/her judgments between all possible pairs of functional and nonfunctional requirements in a similar way as the IPA approach.  Figure 3 shows a snapshot of the TIPA graphical user interface.

The tool supports the user in the whole elicitation process. In particular, after the authentication, TIPA presents the user with an agenda of elicitations. The user can analyze the description of requirements for each pair (i.e., FR and NFR) and specify the preference value in terms of the importance degree of every nonfunctional requirement for achieving each functional requirement, by selecting one of the radio buttons indicated in Figure 3. When the user clicks “Submit,” the next pair of requirements is displayed. Finally, once all the evaluations have been performed, the system is able to compute and show the prioritized list of functional requirements, nonfunctional requirements, and their respective priority values.

The tool was developed in Microsoft Visual Studio 2008 and. NET Framework 3.5 using C*♯* programming language to implement the IPA algorithm. Furthermore, for backing-up the data generated by TIPA, Microsoft SQL Server 2008 R2 was utilized.

## 4. The Experiment

Here, we describe in detail the experiment which we carried out during this study.  Table 6 summarizes the key components of the experiment. They are characterized in terms of the main goal of the experiment, independent variables, dependent variables, empirical evaluation approach, and other factors, following empirical study terminology [29].

The main goal of this experiment is to investigate the actual time-consumption property while using the IPA (see Section 4.1 for more details). Moreover, for the comparative evaluations, we also introduce two other prioritization approaches: an AHP-based prioritization approach (simply refer to AHP in the rest of the paper) and hybrid assessment method (HAM from now on). The former is described in Section 4.2 whereas the latter is illustrated in Section 4.3. The purpose of the comparative evaluations is to find out the answers to the following questions:

RQ1: which approach between IPA and AHP-based approach is less time-consuming in performing the whole prioritization task?
RQ2: are ranks obtained by IPA and AHP similar?
RQ3: which approach between IPA and HAM is less time-consuming in performing the whole prioritization task?
RQ4: are ranks obtained by IPA and HAM similar?

The ultimate goal is to collect evidence whether IPA outperforms the two state-of-the-art-based approaches with respect to the actual time-consumption property.

### 4.1. Measuring Properties

To perform an effective assessment of a requirement prioritization approach, a key issue is to decide which approches' properties need to be measured [30] as well as how to measure them. In our study, time-consumption property is evaluated while using the IPA, AHP, and HAM. Following the definition presented by Perini et al. [30], the time-consumption of a prioritization process is defined as the time interval between the time we get the final ranking (end time of the prioritization task) and the time the user starts prioritizing (start time of the prioritization task). Time-consumption can be measured by monitoring start and end time automatically and then compute their difference (we call it actual time-consumption). Computing the actual time-consumption is useful for answering the research questions, RQ1 and RQ3.

Furthermore, in order to have a measure of the difference between the rankings produced by IPA and the rankings generated by AHP as well as HAM, we use the agreement measure which has been proposed in [31]. This measure is defined as the ratio of the number of common requirements in the first *k* positions of the two ranks *r*_1_ and *r*_2_ over the number of positions, that is, *k*. It should be noted that the relative order of the common requirements in the ranks is not taken into consideration. In other words, if ncr_*r*_1_,*r*_2__(*k*) is the number of common requirements in the two ranks from the first to the *k*th position, the agreement at position *k* is defined as agreement_*r*_1_,*r*_2__(*k*) = ncr_*r*_1_,*r*_2__(*k*)/*k* (e.g., considering two ranks *r*_1_:  *a* > *b* > *c* > *d* and *r*_2_:  *b* > *a* > *d* > *c*, the agreement at 3th position is agreement_*r*_1_,*r*_2__(3) = 0.66). Notice that agreement_*r*_1_,*r*_2__(*k*) = 1.0 means that the same set of requirements has been ordered in the first *k* positions by two ranks, but these requirements may appear in a different relative order in the two rankings. Evaluating this measure can help us to find out the answers to research questions, RQ2 and RQ4.

### 4.2. An AHP-Based Prioritization Approach

To be able to perform a comparative evaluation of IPA, we present here another prioritization approach which aims to prioritize functional requirements and nonfunctional requirements separately using a state-of-the-art method such as AHP [17]. AHP method has been applied in our study since it plays a pivotal role in empirical evaluations of prioritization methods; its properties have been studied deeply, and it has been widely considered as a reference method in several studies in software engineering domain.

The analytic hierarchy process (AHP) [17] is a multiple criteria decision-making method exploited pairwise comparison strategy, allowing comparison of all the possible pairs of requirements together to determine the priority level of one requirement over another requirement. Given a set of *n* requirements specified by a decision maker, the first step in AHP is to construct an *n* × *n* matrix whose rows and columns represent the candidate requirements. Then, the decision maker specifies his/her judgment to each pair of requirements by assigning a preference value which is between 1 and 9, where 1 expresses the equal value while 9 indicates extreme value. In fact, the decision maker has to perform *n*∗(*n* − 1)/2 pairwise comparisons totally. The fundamental values used for this purpose are presented in Table 7, which represents a qualitative measure of preference relation between a given pair of requirements. Once all the pairs have been assessed, a ranking is calculated through the computation of the principal eigenvector of the matrix (i.e., the eigenvector with the highest eigenvalue norm). Each element of the principal eigenvector represents the rank of each requirement.

In our case, to obtain the prioritized lists of functional and nonfunctional requirements, two tasks should be performed separately, applying the AHP method on the candidate list of functional requirements and also applying the AHP method on the candidate list of nonfunctional requirements.

#### 4.2.1. Tool Support

**Figure 4:**
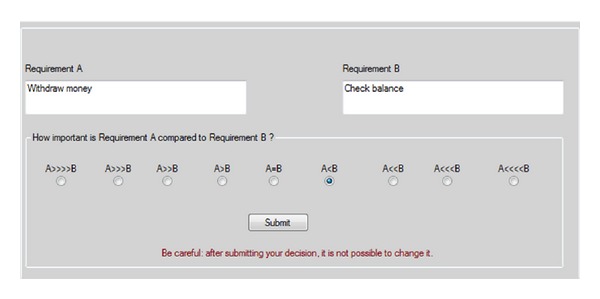
A snapshot of the graphical user interface showing a pair of requirements (withdraw money, check balance) under evaluation in CAHP.

This section describes the tool that was developed within our study for applying the AHP method named Csharp analytic hierarchy process (CAHP) which allows automating the steps of the AHP method described in Section 4.2. The tool guides the user to apply pairwise comparisons between all possible pairs of requirements.  Figure 4 indicates a snapshot of the CAHP graphical user interface where the user can choose the value of the relative importance between the two requirements.

The tool supports the whole evaluation process. In particular, after the authentication, the tool presents the user with an agenda of *n*∗(*n* − 1)/2 pairwise comparisons. The user can analyze the description for each pair of requirements and specify the value of his/her preference in terms of the relative importance of one requirement with respect to the other, by selecting one of the radio buttons shown in Figure 4. When the user clicks “Submit,” the next pair of requirements is displayed. Finally, once the user completed all the evaluations, the system computes the final ranking of the requirements along with their priority values using AHP algorithm.

Analogously to TIPA, the CAHP was developed in Microsoft Visual Studio 2008 and  .NET Framework 3.5 using C*♯* programming language to implement the AHP algorithm. For backing-up the data generated by the tool, Microsoft SQL Server 2008 R2 was applied.

### 4.3. Hybrid Assessment Method (HAM)

In this section, we describe briefly the hybrid assessment method (HAM), proposed by Ribeiro et al. [32], which is used in our study in order to be compared with IPA.

HAM is an integrated multiple criteria decision-making method that combines one pairwise comparison matrix with one classical multicriteria decision matrix to support the process of prioritizing a set of alternatives based on a set of criteria. In fact, HAM is a two-phase method which begins the prioritization task by eliciting the criteria and the alternatives. The second step performs trade-offs between criteria using pairwise comparisons. The third step is to calculate the criteria priority vector, normalize the respective weights, and calculate the consistency ratio. These three steps belong to Phase 1 of the HAM and correspond to an automated determination of weights for the decision matrix. Phase 2 starts in step four, which elicits the contributions of each alternative with respect to each criterion, using a classical weighted average decision matrix. The fifth and final step is an aggregation process to determine the prioritization of alternatives (ranking) using a geometric aggregation operator. This step concludes HAM's process by providing the ratings for each alternative. Interested readers may refer to [32] for more details.

In this study, we used the HAM with the goal of integrating the prioritization of functional and nonfunctional requirements. In such case, we consider nonfunctional requirements and functional requirements as the criteria and the alternatives of HAM's process, respectively. Therefore, Phase 1 of HAM's process is applied in order to obtain the prioritized list of nonfunctional requirements while the second phase is performed to produce the prioritized list of functional requirements. The following section illustrates the tool that implements the desired tasks.

#### 4.3.1. Tool Support

**Figure 5:**
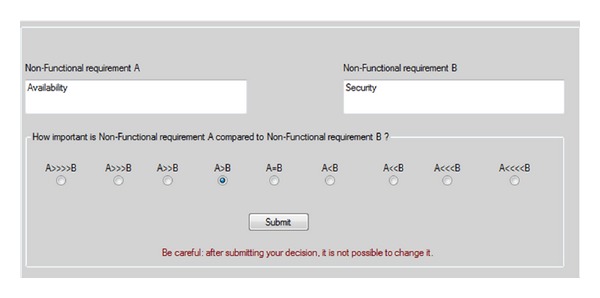
A snapshot of the graphical user interface showing a pair of nonfunctional requirements (availability, security) under evaluation in CHAM.

**Figure 6:**
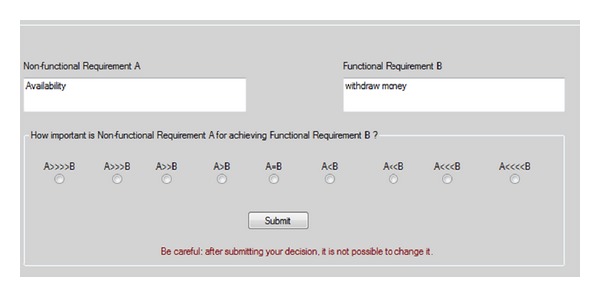
A snapshot of the graphical user interface showing a pair of nonfunctional and functional requirements (availability, withdraw money) under evaluation in CHAM.

This section explains the software tool that was developed within our research for implementing the HAM method, namely, Csharp hybrid assessment method (CHAM), aiming at automating the steps of the HAM method explained in Section 4.3. The tool assists the user to apply pairwise comparisons between all possible pairs of nonfunctional requirements as well as determining his/her judgments between all possible pairs of functional and nonfunctional requirements in a similar way as the HAM method. The tool's main windows are displayed in Figures 5 and 6.

The tool supports the whole prioritization process. In particular, after the authentication, CHAM displays to the user an agenda of pairwise comparisons of nonfunctional requirements (see Figure 5). The user can analyze the description for each pair of nonfunctional requirements and specify the value of his/her preference in terms of the relative importance of one nonfunctional requirement in comparison with the other, by selecting one of the radio buttons shown in Figure 5. When the user clicks “Submit,” the next pair of requirements is displayed. Finally, once the user completed all the evaluations, the system computes the final ranking of the nonfunctional requirements along with their priority values using HAM method. In fact, at this point, Phase 1 of HAM's process is finished and the process for Phase 2 is started by presenting the user with an agenda of determining the importance degree of every nonfunctional requirement for achieving each functional requirement (see Figure 6). Finally, once all the evaluations have been carried out, Phase 2 of HAM's process is terminated and the system is capable of computing the prioritized list of functional requirements according to the HAM method.

The tool was developed in Microsoft Visual Studio 2008 and  .NET Framework 3.5 using C*♯* programming language to implement the HAM algorithm. Furthermore, for backing-up the data generated by CHAM, Microsoft SQL Server 2008 R2 was utilized.

### 4.4. Experiment Definition

We are interested in giving experimental evidence of the capability of the IPA to reduce the actual time-consumption of the prioritization task while preserving the results quality.

For this purpose, during this study, we carried out the experiment aims at investigating the actual time-consumption property of the proposed approach, IPA, in comparison with the other two state-of-the-art-based approaches such as AHP and HAM. Moreover, to analyze the quality of the results produced as output by the IPA approach, we compared IPA's results (i.e., the prioritized lists of functional and nonfunctional requirements) with the results obtained by AHP and HAM. To achieve the desired goals, we evaluated two variables called actual time-consumption and results quality throughout this experiment. To evaluate the actual time-consumption property, we monitored automatically the start time as well as the end time of the prioritization task while using the IPA, AHP, and HAM. In addition, in order to assess the quality of the results produced by IPA in comparison with AHP and HAM, we used the agreement measure which has been described in Section 4.1.

The outline of the experiment is given in the following. The twenty requirements used during our experiment were the requirements of a simulated banking software system, which were divided into two different categories: 15 functional requirements and 5 nonfunctional requirements. These requirements were high level and rather independent requirements that have been selected taking into consideration that they have to be clear enough even for novice users. All requirements were represented as simple textual descriptions. The prioritization was performed without taking into account dependencies among requirements. The subject involved in the experiment is a researcher who has a Ph.D. degree with experience in industrial and research projects and currently has been served as Postdoc at Wisma R&D, University of Malaya, Kuala Lumpur, Malaysia. He has also a good knowledge about different types of software requirements, programming languages, and software engineering domain since he has got his B.S., M.S., and Ph.D. degrees in software engineering. So, we believe that the selected subject for our experiment can be considered not far from industrial developer/analyst.

### 4.5. Experiment Execution

Before running the experiment, we provided a brief presentation to the subject who participated in our experiment with the purpose of giving an introduction on the requirements, prioritization problem and on available methods, IPA, AHP, and HAM, as well as the simulated banking software system. Examples of functional and nonfunctional requirements of the banking software system were explained in order to clarify them. Moreover, we provided a short training on the tools, TIPA, CAHP, and CHAM. Those tools were also tested on a small set of requirements.

The experiment took place in a research laboratory room equipped with computers. A computer with access to TIPA, CAHP, and CHAM has been provided to the subject. The twenty requirements, including 15 functional requirements and 5 nonfunctional requirements, were inserted in advance to the software tools. Given the same set of functional and nonfunctional requirements, the subject executed the three prioritization tasks sequentially. The order of executions was assigned randomly in order to minimize the effect of the order. The experiment took more than 2 hours, including introductory presentation and training on the tools, as well as the short break of 8 minutes between the two prioritization tasks.

### 4.6. Experiment Results

This section describes the main results obtained from the experiment we conducted. The analysis was performed using Microsoft Excel.

**Figure 7:**
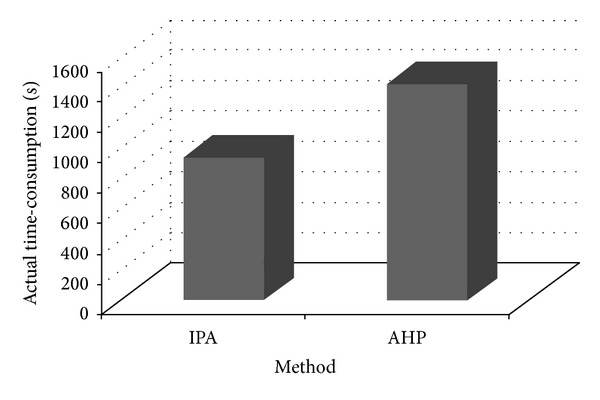
The actual time-consumption for each prioritization task, derived from start/end times recording automatically by the prioritization tools.

*RQ1: Which Approach between IPA and AHP-Based* *Approach Is Less Time-Consuming in Performing the Whole Prioritization Task? *Some insights can be obtained by looking at the results indicated in Table 8 and the chart sketched in Figure 7, which compares the actual time-consumption to perform the prioritization task using IPA and AHP, resulting from start/end times recorded automatically by the prioritization tools. It is clear that the time needed to perform the prioritization task is smaller with IPA than with AHP. As Table 8 shows, the difference in time between the two methods is 481 seconds which corresponds to a reduction of 34%. This is also shown in Figure 7 where the difference in time between the two methods is remarkable.

**Figure 8:**
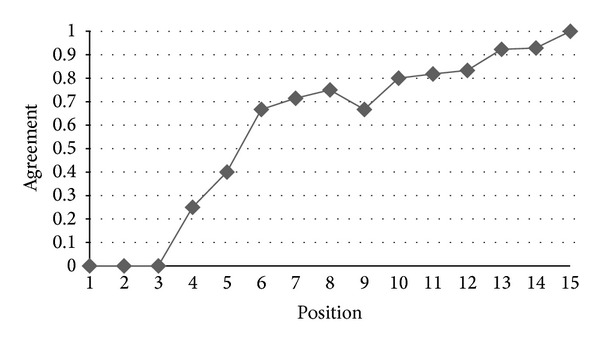
Agreement between the IPA and AHP rankings of functional requirements.

**Figure 9:**
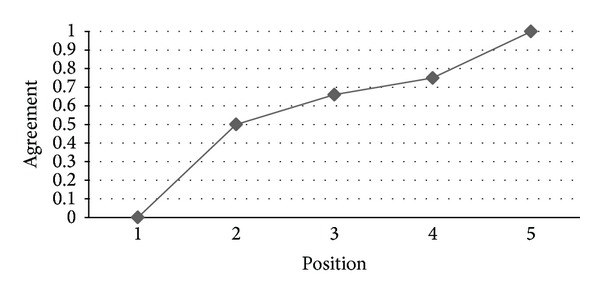
Agreement between the IPA and AHP rankings of nonfunctional requirements.

*RQ2: Are Ranks Obtained by IPA and AHP Similar?* In order to have a measure of the difference between the rankings produced by the IPA and the rankings generated by AHP, we used the agreement measure which has been introduced in Section 4.1.  Figure 8 indicates the agreement level of IPA and AHP for the prioritized list of functional requirements produced by these two methods. The horizontal axis represents the positions of functional requirements in the ranking while the vertical axis shows the value of the agreement of the functional requirements rankings generated by the two methods. As can be seen in Figure 8, there is a good agreement between the two ranks. For example, at the 6th position of the two ranks, we have a very high agreement, close to 0.7, that confirms the results from the analysis of the different ranks. This value increases to 1.0 for higher positions.  Figure 9 shows the same analysis regarding the rankings of nonfunctional requirements produced by these two methods. There is also a good agreement between the two ranks: they have an agreement of 0.5 for the second position which increases to 1.0 for higher positions. 

**Figure 10:**
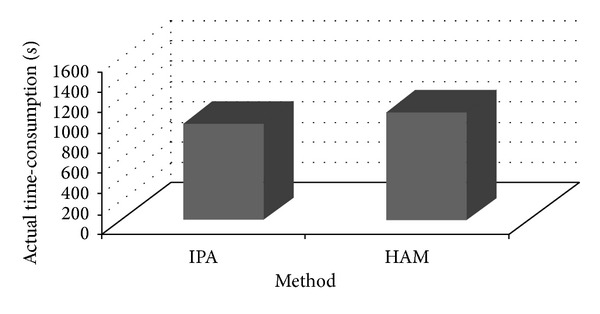
The actual time-consumption for each prioritization task, derived from start/end times recording automatically by TIPA and CHAM.

*RQ3: Which Approach between IPA and HAM Is Less Time-Consuming in Performing the Whole Prioritization Task?* The valuable information regarding the comparison between IPA and HAM in terms of the actual time-consumption of the whole prioritization task can be found easily by looking at Table 9 and Figure 10. We can figure out that the time required to complete the prioritization task is smaller with IPA than with HAM since the difference in time between the two methods is 111 seconds which corresponds to a reduction of 11%. This can also be observed in Figure 10 where the difference in time between the two methods is evident. 

**Figure 11:**
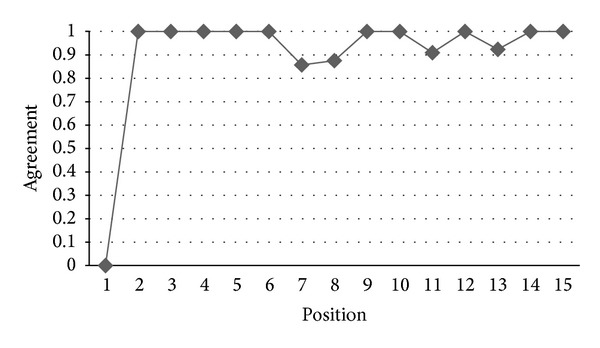
Agreement between the IPA and HAM rankings of functional requirements.

**Figure 12:**
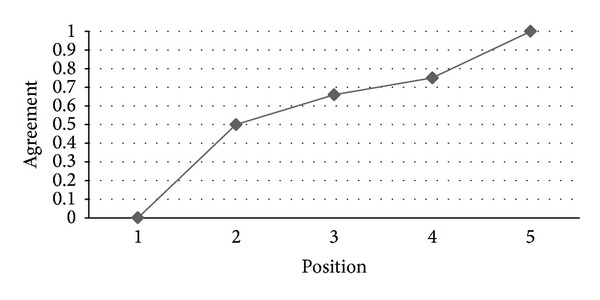
Agreement between the IPA and HAM rankings of nonfunctional requirements.

*RQ4: Are Ranks Obtained by IPA and HAM Similar?* As can be seen in Figure 11, there is a high level of agreement between IPA and HAM for the prioritized list of functional requirements generated by these two methods where we can find that, for the 93% of the positions, the minimum value of the agreement is 0.85 (i.e., at 7th position) while the maximum value is 1.0 which indicates that the similarity between the results obtained by the IPA and HAM for prioritizing functional requirements is remarkable. Analogously to the results obtained for RQ2, there is also a good agreement level between IPA and HAM, as depicted in Figure 12, for the prioritized list of nonfunctional requirements produced by these two methods. In this case, the agreement value is 0.5 for the second position while it increases to 1.0 for higher positions.

### 4.7. Discussion

The observations concerning the actual time-consumption of the three prioritization approaches, IPA, AHP, and HAM, indicate that our proposed approach, IPA, outperforms the other two state-of-the-art-based approaches while at the same time the analysis of the agreement of the rankings produced by these approaches for both functional and nonfunctional requirements shows that IPA preserves the quality of the results obtained by this approach at a high level of agreement in comparison with the results produced by the other two approaches (i.e., AHP and HAM). Regarding the actual time-consumption, the difference between the three approaches seems to depend mainly on the number of comparisons requested to the subject by these approaches where it is 115 for AHP, 85 for HAM, and 75 for IPA. Furthermore, the different range of the values used for expressing a preference applied by the three approaches could have contributed to this difference in time-consumption.

As a final observation, the results point out that IPA should be preferred to AHP and HAM in the prioritization problems with the following characteristics: in those problems, the prioritization of both functional and nonfunctional requirements is needed early in the life cycle meanwhile the time-consumption is a main issue within those problems for performing the prioritization tasks.

We believe that the analysis of the specific results for actual time-consumption and the level of agreement may be used as a pilot study for identifying trends before conducting a study in industry since it gives useful information to guide the selection of the most appropriate approach when deciding which prioritization approach to use for a given project in an organization.

## 5. Conclusions

In this paper, we presented a detailed description of the IPA approach for software requirements prioritization.

The software engineering community has been criticizing the lack of an approach which enables practitioners to integrate the prioritization of functional and nonfunctional requirements [16, 21]. We believe that the approach introduced in this paper is a useful first step toward filling this need. The IPA allows the practitioners to prioritize both functional and nonfunctional requirements simultaneously, producing the prioritized lists of functional and nonfunctional requirements separately. The main contribution of the IPA over existing work is to provide a requirement prioritization framework which considers both functional and nonfunctional requirements jointly during the prioritization process. The results of applying the IPA for a given software project, which are an ordered list of functional requirements as well as a prioritized list of nonfunctional requirements, may assist software developers to concentrate on the most important functional requirements as the key component of the implementation phase early in the life cycle rather than later in the life cycle when modifications are often difficult and impractical to accomplish. It can also help software architects to consider the most significant nonfunctional requirements as the main driver to design the system's software architecture and also simplify the selection of suitable guidelines for achieving the desired quality attributes.

The effectiveness of the proposed approach has been evaluated through an empirical experiment aimed at comparing the IPA with the two state-of-the-art-based approaches, AHP and HAM. We focused mainly on two measures: the actual time-consumption and the results quality. The experiment has been conducted on a simulated case study which consists of twenty requirements including 15 functional and 5 nonfunctional requirements. The main conclusion that can be drawn from the results of the experiment is that the IPA is superior to both AHP and HAM regarding the actual time-consumption while the results produced by IPA are very similar to the results obtained by the other two approaches. Although the generalization of the presented experiment to industrial practice is not straightforward, the results are an important basis for the planning of industrial case studies.

We are currently conducting the same experiment with the one performed in this study but with the participation of more subjects to get a larger data set and thereby to find a stronger basis for our conclusions. As part of the future work, it would be of interest to carry out a controlled experiment in a real industrial setting to see how similar are the results with our findings.

## Figures and Tables

**Table 1:** Steps of IPA for integrating the prioritization of FRs and NFRs.

Step number	Description
1	Determine candidate FRs and NFRs
2	Construct the decision matrix
3	Elicit the importance degree of each NFR with respect to each FR
4	Calculate NFRs final ranking with respect to all FRs using triangular fuzzy numbers and alpha cut approach
5	Compute FRs final ranking using weighted average decision matrix and weights determined in Step 4

**Table 2:** IPA nominal scale and IPA actual scale.

IPA nominal scale	IPA actual scale
Very high importance (VHI)	1
High importance (HI)	0.75
Low importance (LI)	0.5
Very low importance (VLI)	0.25
No importance (NI)	0.001

**Table 3:** Weighted average decision matrix for priority assessment of FRs.

NFRs weights	NW_1_	NW_2_	NW_3_	NW_*m*_
	NFR_1_	NFR_2_	NFR_3_	NFR_*m*_
FR_1_	$\overset{´}{D_{11}}$	$\overset{´}{D_{12}}$	$\overset{´}{D_{13}}$	$\overset{´}{D_{1m}}$
FR_2_	$\overset{´}{D_{21}}$	$\overset{´}{D_{22}}$	$\overset{´}{D_{23}}$	$\overset{´}{D_{2m}}$
⋮	⋮	⋮	⋮	⋮
FR_*n*_	$\overset{´}{D_{n1}}$	$\overset{´}{D_{n2}}$	$\overset{´}{D_{n3}}$	$\overset{´}{D_{nm}}$

**Table:** (a)

$\overset{´}{D}=\begin{array}{cccc} & {\text{NFR}}_{1} & {\text{NFR}}_{2} & {\text{NFR}}_{3}\\ {\text{FR}}_{1} & 1 & 0.75 & 0.25\\ {\text{FR}}_{2} & 0.75 & 0.25 & 0.25\\ {\text{FR}}_{3} & 0.5 & 1 & 0.75\\ {\text{FR}}_{4} & 0.75 & 1 & 0.75 \end{array}$	

**Table:** (b)

	${\tilde{F}}_{x}$	*W*	NW
NFR_1_	(*0.5, 0.728, 1*)	*0.74 *	*0.4 *(*1*)
NFR_2_	(*0.25, 0.6588, 1*)	*0.64 *	*0.346 *(*2*)
NFR_3_	(*0.25, 0.433, 0.75*)	*0.47 *	*0.254 *(*3*)

**Table 5:** Example of computing FRs priority vector.

NFRs weights	0.4	0.346	0.254	*R*	NR
	NFR_1_	NFR_2_	NFR_3_		
FR_1_	1	0.75	0.25	0.637	0.249 (3)
FR_2_	0.75	0.25	0.25	0.388	0.152 (4)
FR_3_	0.5	1	0.75	0.704	0.275 (2)
FR_4_	0.75	1	0.75	0.828	0.324 (1)

**Table 6:** Overview of empirical assessment performed on IPA.

Goal	Analyze the actual time-consumption property of three different prioritization approaches: IPA, AHP-based, and HAM
Independent variable	Prioritization approaches: IPA; AHP-based, and HAM
Dependent variable and measure	Actual time-consumption (measured as computing the difference between start and end time of the prioritization task); results quality (measured in terms of agreement)
Empirical study approach	Simulated case study with real subject: banking software system; 20 requirements including 15 functional requirements and 5 nonfunctional requirements

**Table 7:** Possible scales used for AHP's pairwise comparison [1].

Relative intensity	Definition	Explanation
1	Of equal value	Two requirements are of equal value
3	Slightly more value	Experience slightly favors one requirement over another
5	Essential or strong value	Experience strongly favors one requirement over another
7	Very strong value	A requirement is strongly favored and its dominance is demonstrated in practice
9	Extreme value	The evidence favoring one over another is of the highest possible order of affirmation
2,4, 6,8	Intermediate values between two adjacent judgments	When comprise is needed
Reciprocals	If requirement *i* has one of the above numbers assigned to it when compared with requirement *j*, then *j* has the reciprocal value when compared with *i *	

**Table 8:** Actual time-consumption for the prioritization.

	IPA	AHP	Difference (AHP − IPA)
Actual time-consumption	944 sec (15.7 min)	1425 sec (23.7 min)	481 sec (8 min)
%	—	—	34%

**Table 9:** Actual time-consumption for the prioritization.

	IPA	HAM	Difference (HAM − IPA)
Actual time-consumption	944 sec (15.7 min)	1055 sec (17.5 min)	111 sec (1.8 min)
%	—	—	11%
